# Anemia, from Depraved Conditions of General Nutrition

**Published:** 1907-04

**Authors:** Bichard Ray

**Affiliations:** Kansas City, Mo.


### Anemia, From Depraved Conditions of General Nutrition.

BY RICHARD RAY, M. D., PH. D., KANSAS CITY, MO.

The nutrition of the body, by which we understand the maintenance of its parts, in a fit state to perform their functions, depends upon two main factors—the supply of suitable food, and the assimilation of the same. When either of these factors are disturbed, disorders of nutrition result.

If the food be inadequate or unsuitable, other things being normal, general atrophy will be the consequence, and the same results will evidently follow if the organs of assimilation are at fault.

The result of a deficiency of suitable food, or of faulty assimilation is a general state of mal-nutrition, in which any hereditary tendencies which may exist have a more favorable field for development.

There is a general diminution in the weight of the body, and an imperfect performance of its functions, as is indicated by muscular weakness, mental lassitude, etc., and as the quantity and quality of the blood supply depends largely upon the digestion and assimilation of proper and nourishing foods, we can readily understand why anemia is so closely associated with almost all cases of disorders of nutrition, for one of the chief causes of anemia is that the supply of blood to the body is insufficient, and except in hemorrhage this insufficiency is most generally the result of derangements of elimination.

The majority of the cases of anemia that are regarded and treated as such, fall into the class to which the name of idiopathic has been applied. In such cases the anemic condition is due, not to any disease, so-called, but to disturbances of nutrition generally; that is, of a healthy relation between the demands of the system and the supply of nutrient material, and the proper assimilation of the same.

This condition occurs most frequently in children and in young women at the period of bodily growth, and of the development and early activity of the sexual functions, and when, as is so frequently the case, the air, light, food, occupation and moral relations of the individual are more or less unhealthy.

Where the etiology of anemia is not complex, and where it is classed as idiopathic, as in the growing child, the girl at puberty, or in cases of depraved condition of general nutrition which are not symptomatic of some more grave condition, such as Bright's disease, phthisis, etc., the rational treatment suggests itself, viz.: to bring the alimentary tract and organs of sanguification into a healthy state, and then proceed to build up the general system, and increase the blood supply.

Dyspepsia and constipation require immediate attention. The food must be carefully selected so that it shall not only supply the albuminous elements that are especially deficient in the blood, but be retained and absorbed, and must therefore be both nourishing and digestible.

The patient must also be placed upon nutritive, reconstructive, tonic treatment, and in these cases I find nothing that gives me better results than cod liver oil, more especially when it is combined with the hypophosphites of lime and soda, as in Hagee's Ext. Ol. Morrhuae, which I use.

---

Opsonins and Opsonic Index.—The medical profession is growing wild once more. A new craze has taken hold of us. Almost every other journal contains either communications or editorials upon the Opsonins and Opsonic Index. Professors are devoting series of lectures to the subject, promising to devote more time. One esteemed and venerable old gentleman for whom we have the most profound respect and admiration, predicts that the time is not far distant when drugs will no longer be used, but we will simply get the Opsonic index of a patient, administer the dead bacteria at the proper times, and behold! your patient is a well man again. Of course, he is a therapeutic nihilist and an ultra enthusiast.

But, indeed, from a scientific standpoint it is quite a medical advancement. Wright of London, the father of the idea, has been doing some excellent work along this line for some time. Only recently has it emerged from its experimental stage, to be seized upon eagerly by those truly devoted to medical advancement. Those who have given the subject fair, calm and deliberate consideration recognize it just now as merely something extremely interesting and of a great deal of practical assistance in certain conditions, when the doctor is in reach of an expert laboratory man. A doctor capable of expressing himself upon the subject said that he believed that only those giving their whole time to laboratory work would be capable of making accurate investigations. Perhaps in time some nobleman of the profession might find a method practical for all, as in the Widal reaction; but until then this new medical tool must remain in the hands of a fortunate few.

And what is this so-called opsonic index? It is simply a wedging together, as it were, the old theory of phagocytosis and the more modern theory of immunity. *Practical Medicine,* published at Delhi, India, gives such a clear explanation of the subject that we shall take the liberty of abstracting bodily:

"It is generally known that the white corpuscles have the power to engulf bacilli which come in contact with the blood, the engulfed bacilli being carried to the liver, where they are probably destroyed. Consequently it was thought that by improving the tone, so to speak, of the white corpuscles the power to resist disease was increased. It has recently been found, however, that the phagocytes only have the power to act on bacilli which have been under the influence of something else. Further, it has been conclusively proved that this 'something else' is contained in the serum. The matter which exerts this influence has not been isolated, but for the sake of convenience the name 'opsonings' (a word of Greek derivation meaning feast-providers) has been given to it. On the extent to which these 'opsonings' are present in the blood serum depends the power of a person to resist disease. By bringing the serum obtained from the blood of any person in contact with a bacterial culture of known activity the patient's power of resistance to consumption and other diseases can be measured. The figure which denotes this power of resistance is known as the opsonic index; if the opsonic index be subnormal it can be raised, and with it the patient's power of resistance. The method of raising the opsonic index is interesting. Whenever a hostile force—such, for instance, as bacteria—comes in contact with the blood certain bodies which the blood contains at once become active and oppose the invading force. If the bacteria are more powerful than the defending force, the system is naturally subjected to the ravages of the bacteria. It is a curious fact that the defending force is not able to distinguish between active bacteria and dead bacteria, and, consequently, even when dead bacteria come in contact with the blood, the defending force, being deceived, is at once up in arms and active; but in this case the energy is not wasted in fighting, and is available for resisting other attacks. It is by taking advantage of this phenomenon that the opsonic index is raised into the blood of a patient whose index is subnormal; emulsion of tuberculin — in other words, dead tubercle bacilli—is injected, with the result that the opsonins become more active and the power of resistance is raised. The index, however, is not raised immediately; as a matter of fact, slightly at first and then begins to rise. After rising to a certain point it becomes stationary, and then another dose of tuberculin is administered."

As one can readily observe, great possibilities are promised by this new method. Practical experiments have demonstrated its great usefulness. A case of lupus which had resisted the ordinary treatments, rays, etc., for some time was promptly cured by administration of tuberculin at the proper time, obtained through the opsonic index. Acne, septicemia and other conditions have yielded so promptly to treatment with the use of the index as to probably fill the investigators with well nigh hilarious exultation.

The work thus far gives us great encouragement, but it will be some time before it can be used generally.—*J. M. Bodenheimer, Editorial in The Medical Recorder, Shreveport, La.*

